# Lipoprotein Ratios: A Potential Biomarker for Clinical Diagnosis of Atherosclerosis in Type 1 Diabetic Patients With Foot Ulceration

**DOI:** 10.7759/cureus.14064

**Published:** 2021-03-23

**Authors:** Walid Hassene Hamri, Mustapha Diaf

**Affiliations:** 1 Department of Biology, Djillali Liabes University, Sidi Bel Abbes, DZA

**Keywords:** lipoprotein ratios, atherosclerosis, cardiovascular diseases, diabetic foot ulceration, type 1 diabetes

## Abstract

Background

Lipoprotein ratios are indicators of atherosclerosis and related diseases such as cardiovascular diseases (CVDs). Early and accurate diagnosis of atherosclerotic disease in patients with diabetic foot ulceration (DFU) is required urgently and remains fundamental to assess the risk of CVDs. This study aimed to determine whether lipoprotein ratios can predict atherosclerosis in type 1 diabetic patients with DFU.

Methodology

This was a cross-sectional study including 255 patients with confirmed type 1 diabetes with a male-to-female ratio of 1.19. Patients admitted to the hospital due to diabetes-related complications were divided into the following groups: patients without DFU (n = 153) and patients with DFU (n = 102). Clinical, biological, and pathophysiological features of patients were compared.

Results

Our study reported a distinct predominance of males (54.50%), with a mean age of 28.64 ± 10.92 years and duration of diabetes of 10.40 ± 9.25 years. The prevalence of DFU was 40.0%. The receiver operator characteristic curve was applied to define the best cut-off lipid ratios to detect atherosclerosis. Total cholesterol (TC)/high-density lipoprotein (HDL) ratio was a valid marker for atherosclerosis with a sensitivity of 86.3%, specificity of 71.4%, and diagnostic accuracy of 0.836%. The findings showed that the fourth quartiles (odds ratio [OR] = 83.02 [22.18-310.75]; p = <10^-3^) of TC/HDL ratio was significantly higher in patients with DFU. Similarly, the last quartiles (fourth) of low-density lipoprotein (LDL)/HDL and triglyceride (TG)/HDL ratio were higher in DFU group (OR = 33.71 [12.04-94.38], p = <10^-3^; OR = 9.60 [4.27-21.58], p = <10^-3^; respectively). In the DFU group, conventional lipid profiles and lipid ratios were markedly higher in males compared to females patients (TG = 1.31 ± 0.69 g/L vs. 1.04 ± 0.84 g/L, p = 0.04; respectively; TC/HDL = 4.79 ± 1.04 vs. 4.22 ± 0.98, p = 0.03; respectively; LDL/HDL = 2.91 ± 1.13 vs. 2.17 ± 1.28, p = 0.01; respectively; TG/HDL = 3.65 ± 2.53 vs. 2.67 ± 1.94, p = 0.008; respectively).

Conclusions

Elevated atherogenic indices were significantly associated with the atherosclerotic load in patients with DFU, supporting the use of lipid ratios as a biomarker for the diagnosis of atherosclerosis disease in clinical practice in the future.

## Introduction

Diabetic foot complications are contributing to long-term morbidity and mortality among the diabetic population leading to repeated hospitalizations and high treatment costs for the patients and community at large [1]. It is estimated that 24.4% of the entire healthcare expenditure among the diabetic population is related to foot complications [2]. The risk of diabetic foot ulceration (DFU) and amputation among diabetic patients increases by two to four folds with the progression of age and diabetes duration regardless of the type of diabetes [3]. It has also been proven by many longitudinal epidemiological studies that among diabetic patients, the prevalence of foot ulcers is about 15%, and the lifetime incidence of DFU may be up to 25% [4]. Therefore, the main treatment for DFU is ensuring tissue oxygenation by reducing insufficient tissue nourishment induced by increased atherosclerosis and reduced angiogenesis [5].

A prominent feature of type 1 diabetes (T1D) is atherogenic dyslipidemia, characterized by an increase in triglyceride (TG)-rich lipoproteins, a decrease in plasma levels of high-density lipoprotein (HDL) cholesterol (TC), and an increase in the levels of low-density lipoprotein (LDL) particles [6]. Oxidation of LDL is a key process in the early progression of atherosclerotic diseases and T1D complications [7]. The use of plasma and serum biomarkers for the prediction and diagnosis of atherosclerosis can be a noninvasive and widely available resource for clinical management. The atherogenic indices of plasma known as Castelli’s risk index-I (TC/HDL-c), Castelli’s risk index-II (LDL-c/HDL-c), and the logarithmic ratio of TG to HDL-c concentration [8] are indicators of atherosclerosis and related diseases such as cardiovascular dysfunction and peripheral artery disease (PAD) [9-11].

Defined as the atherosclerotic occlusive disease of the lower limbs, PAD is associated with an increased risk of lower extremity amputation and serves as a biomarker for atherothrombosis in cardiovascular beds [12]. Additionally, PAD can cause severe and prolonged disability in diabetic patients [12]. Furthermore, these patients are at the greatest risk for cardiovascular disease (CVD) morbidity compared with diabetic patients who do not have DFU [13]. Hence, focused clinical examination of the feet, particularly palpation of foot pulses, remains fundamental to assess the risk of CVD [14]. Nevertheless, palpation of foot pulses, especially in the presence of DFU, can be unreliable in screening for the presence of PAD in people with diabetes [14].

Thus, to test the concept that T1D patients with DFU have an increased prevalence of atherosclerotic load, as shown using clinical, immunological, and biological measurements such as blood lipid ratios, we investigated the association between these measures and DFU in a group of patients with T1D but without previous CVD.

## Materials and methods

This was a cross-sectional, observational study covering data from January 1, 2013 to December 31, 2019 on patients with T1D who attended the Diabetes-Endocrinology Department of the University Hospital Center in Sidi Bel Abbes, northwestern Algeria. The study sample consisted of 255 T1D patients diagnosed in their pubertal period (according to the World Health Organization’s criteria) who were over age 13 years at the time of the study. Patients admitted to the hospital due to diabetes-related complications were divided into the following groups: patients without DFU (n = 153) and patients with DFU (n = 102). All the files of the patients were reviewed for the following: medical history, other associated diseases, biochemical parameters, and complications of the diabetic disease. All T1D patients aged more than 13 years, who had angiography with an antegrade approach and had plain radiography of the foot, and visited the Diabetes-Endocrinology Department at least one time a year with no history of any CVD were enrolled in the analysis. We excluded around 80 patients due to missing valid information on the disease status and missing informed consent.

For all patients, anthropometry parameters including body weight and height, body mass index (BMI), and waist circumference were available in the patient’s medical record. Blood pressure was measured using a sphygmomanometer in a supine position followed by a second measurement (after a few minutes) in a standing position. Hypertension was defined by systolic blood pressure (SBP) of 140 mmHg and diastolic blood pressure (DBP) of about 90 mmHg or more. The latest biochemical assessment including fasting blood glucose, glycated hemoglobin (HbA1c), hemoglobin (Hb), high-sensitivity C-reactive protein (hs-CRP), urea, serum creatinine, urinary albumin excretion rate (UAER), and lipid parameters, namely, total cholesterol (TC), HDL, LDL, and TG, were measured in the hospital during the period of hospitalization by MINDRAY BS-230 using a commercial kit. All data were taken from patients’ medical records. The ulcers in patients with diabetic foot were graded from 1 to 5 according to the presence of infection and/or gangrene using the Wagner Classification [15] (grade 1: presence of superficial ulcer limited to the epidermis; grade 2: infection reaching the dermis, muscle, tendon, and ligaments but no signs of osteomyelitis; grade 3: presence of deep soft tissue infection and osteomyelitis; grade 4: gangrene localized to the distal foot; grade 5: extensive gangrene). Examination findings and culture and radiology results were utilized to classify the foot ulcers. Moreover, lipid ratios as indicators of atherogenic risk were calculated, namely, TC/HDL, LDL/HDL, and TG/HDL.

Due to the sample size, all data were compiled and analyzed using SPSS version 22 (IBM Corporation, Chicago, IL, USA). The descriptive statistics of mean ± standard deviation with its respective 95% confidence intervals (95% CI) were estimated for quantitative variables, whereas qualitative variables were expressed as percentages (%) and relative frequencies. Continuous variables underlying normal distribution were compared between groups using Student’s t-test, while categorical variables were compared between groups with the Chi-square test. Odds ratios (OR) and 95% CIs for lipid profiles were determined using multivariate logistic regression analysis after adjustment across quartiles of lipid ratios to investigate the association between DFU and atherosclerosis. The receiver operator characteristic (ROC) curve was used to determine the best cutoff value and validity of lipid ratios, and the area under the ROC curve (AUC), sensitivity, specificity, positive predictive value (PPV), and negative predictive value (NPV) were calculated. Statistically significant differences were maintained when the p-value was less than or equal to 0.05.

## Results

The basic characteristics of the studied patients are shown in Table 1. A total of 255 T1D patients (54.50% males and 45.50% females) were enrolled during the period of the study. For the purpose of the analysis, patients were classified into two groups according to the presence of DFU. Of the 255 diabetic patients, 102 (40.00%) patients had DFU. For all patients, the mean age was 28.64 ± 10.92 years (range = 13-70 years), and the mean diabetes duration was 10.40 ± 9.25 years. Meanwhile, the mean age of patients with DFU was significantly higher than those without DFU (33.98 ± 11.98 years vs. 25.07 ± 8.48 years, p = <10^-3^, respectively). Similarly, the mean duration of diabetes was significantly higher among those who developed DFU compared to those who did not (13.55 ± 10.32 years vs. 8.30 ± 7.81 years, p = <10^-3^, respectively) (Table 2). The most affected age group with DFU was the 30-39 years with a rate of 30.40%, followed by the 40-49 years group with a rate of 26.50%. However, the least affected age group was the 13-19 years. Notably, history of smoking showed a slight association with the development of DFU (23.50%, p = 0.08) whereas there were significant differences across the different weight categories (p = <10^-3^) (Table 1).

**Table 1:** Basic characteristics of the study participants. DFU: diabetic foot ulceration; BMI: body mass index Percentages were compared with Chi-square test, p ≤ 0.05 was considered as significant

Variables	All patients (n=255)	Without DFU (n=153)	With DFU (n=102)	P-Value
	Number (%)	Number (%)	Number (%)	
Gender				
Male	139 (54.50%)	75 (49.00%)	64 (62.70%)	0.03
Female	116 (45.50%)	78 (51.00%)	38 (37.30%)	
Age groups				
13-19 years	49 (19.20%)	48 (31.40%)	1 (1.00%)	<10^-3^
20-29 years	79 (31.00%)	67 (43.80%)	12 (11.80%)	
30-39 years	58 (22.70%)	27 (17.60%)	31 (30.40%)	
40-49 years	36 (14.10%)	9 (5.90%)	27 (26.50%)	
50-59 years	30 (11.80%)	2 (1.30%)	28 (27.40%)	
More than 60 years	3 (1.20%)	0 (0.00%)	3 (2.90%)	
Smoking history				
Male	47 (18.40%)	23 (15.00%)	24 (23.50%)	0.08
Prevalence of weight categories				
Underweight, BMI < 18.5 kg/m²	16 (6.30%)	13 (8.50%)	3 (2.90%)	<10^-3^
Normal weight, BMI = 18.5–25.0 kg/m²	144 (56.50%)	83 (54.30%)	61 (59.80%)	
Overweight, BMI = 25.0–29.9 kg/m²	55 (21.60%)	38 (24.80%)	17 (16.70%)	
Obesity, BMI = 30 kg/m²	40 (15.60%)	19 (12.40%)	21 (20.60%)	
Other associated complications				
Low visual acuity	66 (25.90%)	29 (19.00%)	37 (36.30%)	0.002
Diabetic nephropathy	22 (8.60%)	6 (3.90%)	16 (15.70%)	0.001
Hypertension	27 (10.60%)	6 (3.90%)	21 (20.60%)	<10^-3^
Hypothyroidism	19 (7.50%)	14 (9.20%)	5 (4.90%)	0.20
Hyperthyroidism	6 (2.40%)	2 (1.30%)	4 (3.90%)	0.17
Anemia	73 (28.60%)	38 (24.80%)	35 (34.30%)	0.10
Dyslipidemia	6 (2.40%)	1 (0.70%)	5 (4.90%)	0.02
Diabetic retinopathy	46 (18.00%)	18 (11.80%)	28 (27.50%)	0.002
Ketosis on diabetes	93 (36.50%)	43 (28.10%)	50 (49.00%)	<10^-3^
Symptoms and signs				
Weight loss	142 (55.70%)	88 (57.50%)	54 (52.90%)	0.34
Polyuria-polydipsia	238 (90.30%)	142 (92.80%)	96 (94.10%)	0.70
Asthenia	107 (42.00%)	66 (43.10%)	41 (40.20%)	0.50
Overeating	121 (47.50%)	73 (47.70%)	48 (47.00%)	0.90

In the current study, the most common complications in both groups were ketosis on diabetes (36.50%, p = <10^-3^), followed by anemia (28.60%, p = 0.10). In contrast to patients without DFU, low visual acuity, diabetic retinopathy, hypertension, diabetic nephropathy, and dyslipidemia were significantly higher in those with DFU (36.30%, p = <0.002; 27.50%, p = <0.002; 20.60%, p = <10^-3^; 15.70%, p = 0.001; and 4.90%, p = 0.02, respectively) (Table 1).

The clinic characteristics for both groups are summarized in Table 2. Concerning the anthropometric measurement on admission, there were higher significant differences in body weight between both groups (57.87 ± 12.63 kg and 62.11 ± 11.39 kg for patients without DFU and with DFU, p = 0.007, respectively) and BMI (p = <0.01), while there were no significant differences in waist circumference and body height (p = <0.31 and p = <0.15, respectively). In addition, significantly higher SBP (p = <10^-3^) and DBP (p = <10^-3^) was found in patients with DFU compared to those without DFU (Table 2).

Interestingly, similar to fasting plasma glucose concentrations, there was a significant difference in HbA1c levels (patients without DFU: 9.32 ± 2.35%; patients with DFU: 11.38 ± 2.08%, p = <10^-3^) between both groups on admission. No significant difference was found between the patients with and without DFU in terms of Hb (p = 0.37). On the other hand, hs-CRP was markedly higher in patients with DFU than those without DFU (12.33 ± 5.24 mg/L vs. 2.30 ± 1.27 mg/L, p = <10^-3^, respectively). With regard to lipid levels, as shown in Table 2, TC, HDL-c, LDL-c, and TG levels differed significantly among the two groups (p = <10^-3^, p = <0.01, p = <10^-3^, and p = <10^-3^, respectively). Further, it was found that lipid ratios (TC/HDL-c, LDL/HDL-c, and TG/HDL-c) were significantly greater in the group with DFU (p = <10^-3^, p = <10^-3^, and p = <10^-3^, respectively) (Table 2).

**Table 2:** Comparison of clinical characteristics between patients with and without DFU. DFU: diabetic foot ulceration; SD: standard deviation; CI: confidence interval; HbA1c: glycosylated hemoglobin; BMI: body mass index; SBP: systolic blood pressure; DBP: diastolic blood pressure; Hb: Hemoglobin; hs-CRP: high-sensitivity C-reactive protein; TC: total cholesterol; HDL-c: high-density lipoprotein cholesterol; LDL-c: low-density lipoprotein cholesterol; TG: triglycerides Means were compared with independent sample Student’s t-test; a p ≤ 0.05 was considered as significant

Variables	All patients (n=255)		Without DFU (n=153)		With DFU (n=102)		P-Value
	Mean±SD	95% CI	Mean±SD	95% CI	Mean±SD	95% CI	
Mean age (years)	28.64 ± 10.92	27.29-29.98	25.07 ± 8.48	23.72-26.43	33.98 ± 11.98	31.63-36.34	<10^-3^
Diabetes duration (years)	10.40 ± 9.25	9.26-11.54	8.30 ± 7.81	7.05-9.55	13.55 ± 10.32	11.52-15.58	<10^-3^
Age at first diagnosis (years)	18.32 ± 8.21	17.30-19.33	16.88 ± 8.05	15.59-18.16	20.48 ± 8.02	18.90-22.06	0.001
Body height (m)	1.68 ± 0.09	1.67-1.69	1.67 ± 0.09	1.66-1.69	1.69 ± 0.08	1.67-1.71	0.15
Body weight (kg)	59.57 ± 12.31	58.05-61.09	57.87 ± 12.63	55.86-59.89	62.11 ± 11.39	59.87-64.35	0.007
BMI (kg/m²)	20.97 ± 3.92	20.49-21.46	20.50 ± 3.96	19.87-21.14	21.68 ± 3.79	20.93-22.42	0.01
Waist circumference (cm)	81.62 ± 11.03	79.23-84.01	80.45 ± 11.74	76.88-84.03	82.90 ± 10.18	79.64-86.16	0.31
SBP (mmHg)	113.3 ± 12.8	111.8-114.9	110.7 ± 11.3	108.8-112.5	117.4 ± 13.8	114.6-120.1	<10^-3^
DBP (mmHg)	66.9 ± 8.9	65.8-68.0	65.2 ± 8.0	63.9-66.5	69.4 ± 9.7	67.5-71.3	<10^-3^
Fasting plasma glucose (g/L)	2.52 ± 0.96	2.40-2.64	2.11 ± 0.57	2.01-2.20	3.13 ± 1.10	2.92-3.35	<10^-3^
HbA1c (%)	10.55 ± 2.34	10.25-10.84	9.32 ± 2.35	9.11-10.39	11.38 ± 2.08	10.96-11.80	<10^-3^
Hb (g/L)	12.59 ± 2.03	12.30-12.87	12.69 ± 1.82	12.36-13.02	12.43 ± 2.32	11.91-12.95	0.37
hs-CRP (mg/L)	4.99 ± 5.32	3.70-6.29	2.30 ± 1.27	1.94-2.67	12.33 ± 5.24	9.72-14.94	<10^-3^
TC (g/L)	1.55 ± 0.40	1.50-1.60	1.35 ± 0.20	1.32-1.39	1.85 ± 0.45	1.76-1.94	<10^-3^
HDL-c (g/L)	0.45 ± 0.10	0.44-0.46	0.46 ± 0.08	0.45-0.48	0.43 ± 0.13	0.40-0.46	0.01
LDL-c (g/L)	0.91 ± 0.31	0.87-0.95	0.78 ± 0.18	0.75-0.81	1.12 ± 0.36	1.04-1.19	<10^-3^
TG (g/L)	1.00 ± 0.64	0.92-1.08	0.80 ± 0.42	0.73-0.87	1.29 ± 0.79	1.13-1.44	<10^-3^
TC/HDL-c	3.61 ± 1.29	3.45-3.77	2.96 ± 0.58	2.87-3.06	4.58 ± 1.46	4.29-4.87	<10^-3^
LDL/HDL-c	2.16 ± 1.00	2.04-2.29	1.72 ± 0.51	1.64-1.81	2.82 ± 1.20	2.58-3.05	<10^-3^
TG/HDL-c	2.41 ± 1.84	2.18-2.63	1.77 ± 0.98	1.61-1.93	3.36 ± 2.35	2.90-3.82	<10^-3^
Creatinine (mg/L)	12.38 ± 12.14	10.12-14.63	9.70 ± 8.07	7.73-11.66	16.20 ± 15.60	11.61-20.78	0.004
Urea (g/L)	0.40 ± 0.33	0.33-0.46	0.32 ± 0.25	0.26-0.38	0.50 ± 0.39	0.39-0.62	0.003
Microalbuminuria (mg/24 h)	48.37 ± 73.44	35.47-61.28	12.77 ± 10.71	10.27-15.27	96.50 ± 92.59	71.23-121.78	<10^-3^

With respect to renal function, patients with DFU had increased plasma levels of creatinine and urea and microalbuminuria (16.20 ± 15.60 mg/L, p = 0.004; 0.50 ± 0.39 g/L, p = 0.003; and 96.50 ± 92.59 mg/24 h, p = <10^-3^, respectively) (Table 2).

For the entire study population, the multivariate regression between lipid ratios quartiles, in terms of their strong association with atherosclerosis, plotted that the fourth quartiles (OR = 83.02 [22.18-310.75], p = <10^-3^) of TC/HDL ratio was significantly higher in patients with DFU, as described in Table 3. Similarly, the last quartiles (fourth) of LDL/HDL and TG/HDL ratios were higher in DFU group (OR = 33.71 [12.04-94.38], p = <10^-3^; OR = 9.60 [4.27-21.58], p = <10^-3^, respectively) (Table 3).

**Table 3:** Crude OR of blood lipid ratios quartiles associated with DFU. DFU: diabetic foot ulceration; CI, confidence interval; OR, odds ratio; TC: total cholesterol; LDL: low-density lipoprotein cholesterol; HDL: high-density lipoprotein cholesterol; TG: triglycerides Multivariate logistic regression significant at p ≤ 0.05

Variables	Without DFU (n=153)	With DFU (n=102)	OR (95% CI)	P-Value
	Number (%)	Number (%)		
TC/HDL ratio				
First quartile (1.42-2.72)	49 (32.0%)	12 (11.8%)	Reference	---
Second quartile (2.73-3.27)	55 (35.9%)	10 (9.8%)	0.74 [0.29-1.86]	0.52
Third quartile (3.28-4.22)	46 (30.1%)	19 (18.6%)	1.68 [0.73-3.85]	0.21
Fourth quartile (4.23-8.21)	3 (2.0%)	61 (59.8%)	83.02 [22.18-310.75]	<10^-3^
LDL/HDL ratio				
First quartile (0.33-1.45)	48 (31.4%)	14 (13.7%)	Reference	---
Second quartile (1.46-1.91)	53 (34.6%)	13 (12.7%)	0.84 [0.35-1.96]	0.69
Third quartile (1.92-2.56)	46 (30.1%)	16 (15.7%)	1.19 [0.52-2.71]	0.67
Fourth quartile (2.57-5.97)	6 (3.9%)	59 (57.9%)	33.71 [12.04-94.38]	<10^-3^
TG/HDL ratio				
First quartile (0.31-1.23)	48 (31.4%)	16 (15.7%)	Reference	---
Second quartile (1.24-1.88)	52 (34.0%)	12 (11.8%)	0.69 [0.29-1.61]	0.39
Third quartile (1.89-2.79)	38 (24.8%)	26 (25.4%)	2.05 [0.96-4.36]	0.06
Fourth quartile (2.80-14.95)	15 (9.8%)	48 (47.1%)	9.60 [4.27-21.58]	<10^-3^

**Figure 1:**
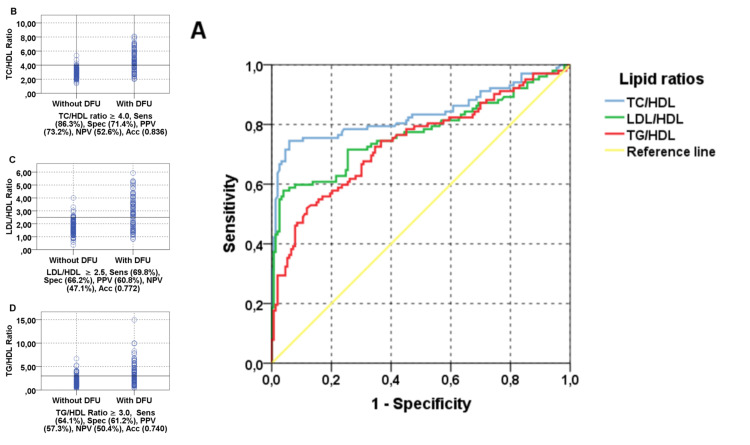
ROC curve to define the best cut-off lipid ratios to detect atherosclerosis. DFU: diabetic foot ulceration; Sens: sensitivity; Spec: specificity; PPV: positive predictive value; NPV: negative predictive value; Acc: accuracy; TC: total cholesterol; LDL: low-density lipoprotein cholesterol; HDL: high-density lipoprotein cholesterol; TG: triglycerides; ROC: receiver operating characteristics

ROC curve was applied to define the best cut-off lipid ratios to detect atherosclerosis. TC/HDL ratio was a valid marker for atherosclerosis. The optimum cut-off value was ≥4.0, with a sensitivity of 86.3%, specificity 71.4%, PPV 73.2%, and NPV 52.6% with a diagnostic accuracy of 0.836. However, LDL/HDL ratio was a moderately valid marker for atherosclerosis. The optimum cut-off value was ≥2.5, with a sensitivity of 69.8%, specificity 66.2%, PPV 60.8%, and NPV 47.1% with a diagnostic accuracy of 0.772. As for TG/HDL ratio, the optimum cut-off value was ≥3.0, with a sensitivity of 64.1%, specificity 61.2%, PPV 57.3%, and NPV 50.4% with a diagnostic accuracy of 0.740 (Figure 1).

**Figure 2:**
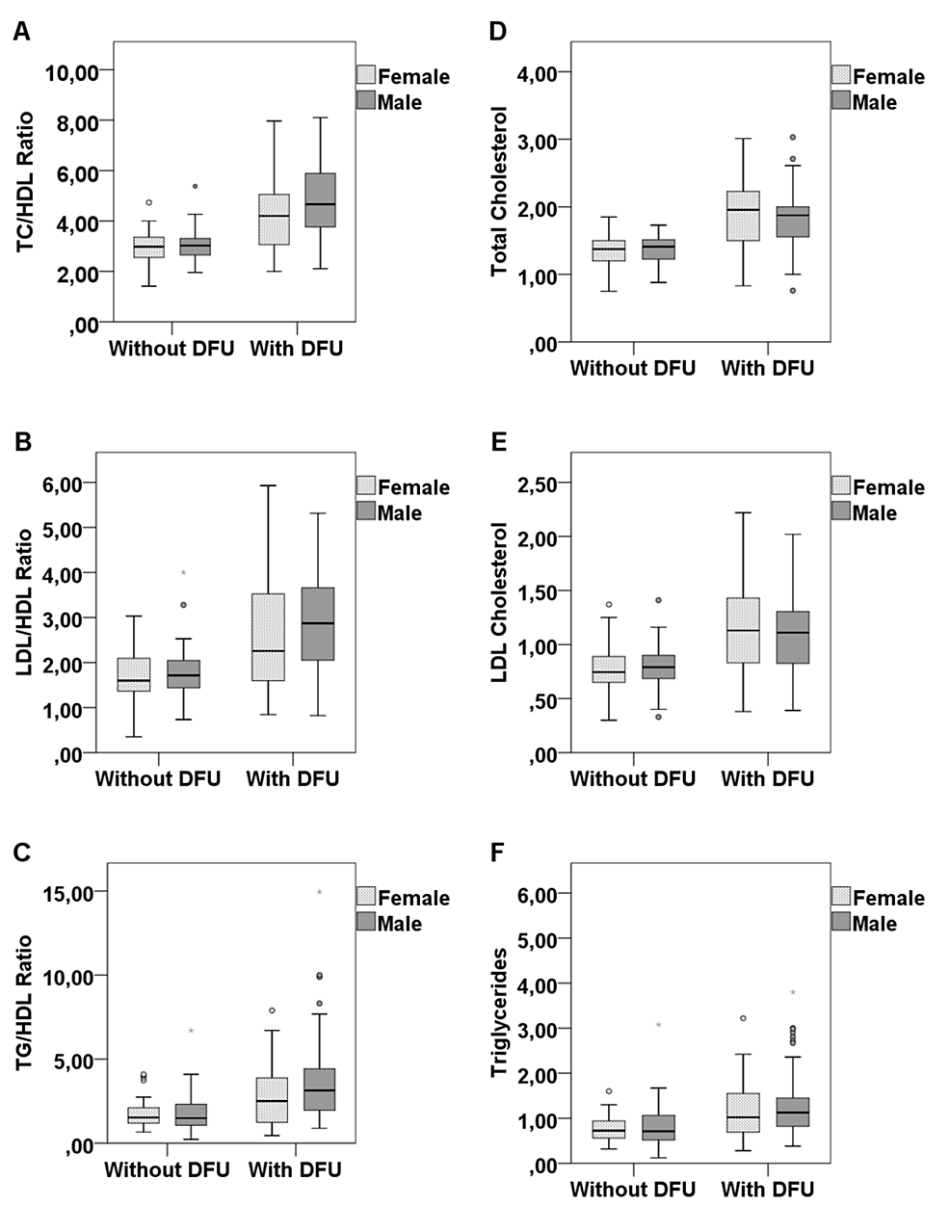
Comparison of lipid ratios levels between patients with and without DFU according to their gender. DFU: diabetic foot ulceration; TC: total cholesterol; LDL: low-density lipoprotein cholesterol; HDL: high-density lipoprotein cholesterol; TG: triglycerides

As shown in Figure 2A-2C, in males and females, when comparing all lipid ratios (TC/HDL, LDL/HDL, TG/HDL) between the two groups, higher levels were found in males compared to females with DFU. As for conventional lipid parameters (TC and LDL), higher values were found in females compared to males with DFU (Figures 2D, 2E).

## Discussion

In this study, we evaluated the potential role of clinical, immunological, and biological features in patients with prolonged duration of T1D with and without DFU to identify the variables linked to the presence of DFU in this population. Interestingly, apart from other well-established CV risk factors, our findings support the theory that macrovascular diseases such as DFU may contribute to the development of CVD in patients with T1D. Thus, we investigated the association between DFU and the occurrence of atherosclerotic disease (via lipid ratios) by comparing two groups of patients according to the presence of DFU (patients without DFU vs. patients with DFU). Our first observation was that the distribution of patients by gender was unequal by demonstrating a distinct predominance of males over females (54.50-45.50%), with a male to female ratio of 1.19. Among patients with DFU (40.0%), the prevalence of foot ulceration was significantly higher in males than in females with T1D. A large retrospective cohort study conducted by Al-Rubeaan et al. [16] showed that 2,071 patients with diabetes had DFU; of those, 1,420 (68.57%) were men; whereas, in the study by Bruun et al., a follow-up study group, 38 (2.8%) patients had DFU at diabetes diagnosis. Of the 38 patients, 27 (71.0%) were men. After a six-year follow-up, 28 (2.9%) had DFU, and of those, 18 (64.3%) were men [17].

Our study displayed significant impacts of age and diabetes duration on the prevalence of DFU. The correlation of age and diabetes duration with DFU risk is similar to studies conducted by several authors, for example, Nehring et al. [18]. According to the study by Al-Maskari, the longer the duration of diabetes, the greater is the risk of developing DFU [19]. In a follow-up study by Al-Delaimy et al., the relative risk (95% CI) for DFU compared with patients without diabetes was (1.39 [0.82-2.36]) for one to five years of diabetes, (3.63 [2.23-5.88]) for six to 10 years of diabetes, (2.55 [1.50-4.32]) for 11 to 25 years of diabetes, and (4.53 [2.39-8.58]) for more than 25 years of diabetes [20]. The present study also shows that cigarette smoking increased the probability of DFU in the entire study population. A similar conclusion was proffered by Al-Rubeaan et al. with reference to patients with type 2 diabetes [16]. In the present study, body corpulence increased the risk of DFU. Nehring et al. investigated the effect of BMI on the progression of DFU and concluded that obese diabetic patients are prone to developing diabetic foot complications [18]. Hurley et al. described that the risk of the diabetic foot doubles when a patient’s weight is higher than the optimal body weight [21]. Diabetic nephropathy and retinopathy increased the prevalence of DFU in the current study, which could be explained by microangiopathic changes [22], which is also the same observation reported in other studies [16,17,19].

In the current study, hypertension was detected in 27 cases, and 21 (20.60%) cases with DFU were hypertensive. Our findings are in line with the previous studies which prove that the prevalence of DFU increases in hypertensive patients [12,19,23]. An important observation of our study was the association of an increased prevalence of DFU with the presence of ketosis in patients with T1D. Our findings echo the observations made by McIntyre who suggested that the avoidance of ketoacidosis may be the key to the prevention of diabetic foot complications [24]. We also observed an incremental increase in HbA1c values in patients with DFU, suggesting a significant relationship between the diabetic foot conditions and the degree of glycemic control. Previous studies reported that poor glycemic control was associated with a two-fold increase in the risk of foot lesions among diabetic patients [25,26].

Our study has shown strong and consistent associations between the amount of hs-CRP and DFU. Statistically significant correlations were shown in a case-control study between DFU and elevated concentrations of hs-CRP [27]. In addition to reduced glomerular filtration rate (as used to define diabetic nephropathy), we further observed a sustained increase in UAER (≥30 mg/24 h) in the DFU group, which may allow these results to be more reliable. Al-Maskari et al. confirmed that the presence of microalbuminuria is a significant risk factor for foot complications [19]. This was in accordance with a previous follow-up study in newly diagnosed diabetic patients observed for 19 years [17]. Additionally, consistent with several previous studies [6,27,28], we also found that there were higher TG concentrations and lower HDL-c concentrations in the DFU group compared with the group without DFU. These observations suggest that lipid ratios can be utilized to evaluate the occurrence of atherosclerosis and the severity of the disease, supporting the employment of this index as an ideal biomarker for diagnosis in clinical practice [28].

Atherosclerosis is known to occur in the proximal arteries of the diabetic limb and presents as iliac, femoral, and popliteal diseases. The risk factor for this proximal site disease is dyslipidemia [29]. Therefore, as a new comprehensive indicator of blood lipid levels, lipid ratios have received increasing attention and can be particularly useful in predicting the risk of CVD [8]. Furthermore, lipid ratios are more reliable than classic lipid parameters in predicting arterial stiffness and contribute significantly to the estimation of CVD risk and arterial stiffness, especially when the absolute values of lipid profile seem normal or not markedly deranged [30].

Despite strong evidence for the utility of lipid ratios in the diagnosis of atherosclerosis, several limitations exist that warrant further study. First, the design of this cross-sectional study was observational and examined a relatively small number of patients because of constrictive inclusion and exclusion criteria. Second, our study highlights only one extremity with diabetic ulcer and gangrene because we restricted it to those with established ulceration where robust exclusion is more important than specificity. Nevertheless, we believe that our study is important from a public health and clinical perspective. To the best of our knowledge, it is the first study that assessed DFU complications in a moderately large sample of T1D in our population. Another strength of this research is the area-specific analysis of DFU risk among the study patients. This study will help address the regional disparities in diabetes foot care in Algeria and in remodeling the chain of diabetes centers where appropriate.

## Conclusions

In conclusion, this study showed that the atherosclerotic load in patients with DFU is significantly higher than in patients without DFU. Likewise, we found that lipid ratios were significantly associated with DFU and can be used as a biomarker for the diagnosis of atherosclerosis disease in clinical practice in the future. Further, based on the findings of this study, we recommend that specialized diabetes care centers should concentrate more on the contributing factors of DFU in the management of patients with T1D as these are associated with morbidity and mortality resulting from CVD.
